# CRISPR/Cas9 mediated genome editing in ES cells and its application for chimeric analysis in mice

**DOI:** 10.1038/srep31666

**Published:** 2016-08-17

**Authors:** Asami Oji, Taichi Noda, Yoshitaka Fujihara, Haruhiko Miyata, Yeon Joo Kim, Masanaga Muto, Kaori Nozawa, Takafumi Matsumura, Ayako Isotani, Masahito Ikawa

**Affiliations:** 1 Research Institute for Microbial Diseases, Osaka University, Suita, Osaka 5650871 Japan; 2 Graduate School of Pharmaceutical Sciences, Osaka University, Suita, Osaka 5650871 Japan; 3 Research Fellow of Japan Society for the Promotion of Science (JSPS), Tokyo 1020083, Japan; 4 Graduate School of Medicine, Osaka University, Suita, Osaka 5650871 Japan; 5 Immunology Frontier Research Center, Osaka University, Suita, Osaka 5650871 Japan

## Abstract

Targeted gene disrupted mice can be efficiently generated by expressing a single guide RNA (sgRNA)/CAS9 complex in the zygote. However, the limited success of complicated genome editing, such as large deletions, point mutations, and knockins, remains to be improved. Further, the mosaicism in founder generations complicates the genotypic and phenotypic analyses in these animals. Here we show that large deletions with two sgRNAs as well as dsDNA-mediated point mutations are efficient in mouse embryonic stem cells (ESCs). The dsDNA-mediated gene knockins are also feasible in ESCs. Finally, we generated chimeric mice with biallelic mutant ESCs for a lethal gene, *Dnajb13*, and analyzed their phenotypes. Not only was the lethal phenotype of hydrocephalus suppressed, but we also found that *Dnajb13* is required for sperm cilia formation. The combination of biallelic genome editing in ESCs and subsequent chimeric analysis provides a useful tool for rapid gene function analysis in the whole organism.

The genome sequencing projects revealed that approximately 20,500 protein-coding genes are encoded in the 3.3 billion base-pairs of DNA sequence in both humans and mice (http://www.genome.gov/12011238). In addition to the protein coding regions, flanking regions play important roles in regulating gene expression. Recent studies also highlighted the importance of non-coding RNAs such as miRNAs for transcriptional and post-transcriptional control^1^,^2^,^3^,^4^. Overall, the genetic sequence of the genome is the blueprint of all living organisms. Gene-manipulated animals have been a mainstay in studying physiological functions. The adaptation of gene targeting in ESCs and subsequent chimeric mouse production and intercrosses enables us to generate homozygous knockout (KO) mice and study gene function in mammals^5^,^6^,^7^. However, only a few laboratories can take advantage of these gene targeting strategies because these experiments are time-consuming, laborious, and costly.

In 2006, the International Knockout Mouse Consortium started to generate a predesigned knockout mouse library to accelerate physiological studies^5^,^6^,^7^. The targeting strategy is well designed so that researchers can observe LacZ reporter gene expression in the heterozygous state, analyze phenotype in the homozygous state, and generate conditional KOs with promoter driven Cre lines in most cases. With this technology, nearly 20,000 protein coding genes have been knocked out in ESCs to date. However, their use is limited by the inefficient germline transmission of the targeted ESCs and the complicated processes of serial mating. More importantly, as we know more about gene features, researchers require a more finely-tuned gene modification system for gene functional analysis as well as making disease model animals. Thus, an efficient system for generating these animals on demand is necessary.

The emergence of the CRISPR/Cas9 system opened a new era for mammalian genome editing^8^,^9^. The sgRNA/CAS9 complex recognizes a target site in the genome by 20 nts upstream of the protospacer adjacent motif (PAM) to cause a blunt end double-strand break (DSB) between 3^rd^ and 4^th^ nucleotides upstream of the PAM. After DSB formation, non-homologous end-joining (NHEJ) causes indels that disrupt gene functions. If a reference DNA strand is co-injected, designed mutations and transgenes are efficiently incorporated by homology-dependent repair (HDR) following DSBs. The CRISPR/Cas9 system works efficiently in mammalian cells and the targeted gene-manipulated mice can be generated by simply injecting sgRNA/CAS9 complex into the pronuclei of fertilized eggs with or without the reference DNA, depending on the desired mutation^10^,^11^,^12^. Therefore, sgRNA/CAS9-mediated genome editing in fertilized eggs is now widely used in many laboratories and animal facilities to generate gene-manipulated mice.

However, some factors remain to be optimized. For targeted gene disruption, small indels are easily introduced by single sgRNA/CAS9 complexes, but removal of the entire gene by two sgRNA/CAS9 complexes becomes less efficient as the distance between the two sgRNAs increases. The efficiency of HDR-mediated gene manipulation in mouse oocytes varies among not only target genes but also among laboratories^12^,^13^,^14^,^15^. More importantly, the mice generated by sgRNA/CAS9 complexes were thought to be homozygous in the founder generation. However, later experiments demonstrated the high prevalence of mosaicism in most founder animals which obfuscate genotyping and phenotyping analyses (Fig. 1)^16^,^17^. Subsequent matings are required to generate fully homozygous mutant animals, making the experimental process similar to conventional ESC-mediated gene manipulation.

In the present study, we examined complicated genome editing in mouse ESCs (Fig. 1). The indels and large deletions were efficiently obtained compared to fertilized eggs. It is noteworthy that while the HDR efficiency with a single-stranded oligodeoxynucleotide (ssODN) is comparable in eggs and ESCs, dsDNA-mediated HDR is more efficient in ESCs. Finally taking advantage of efficient genome editing in ESCs, we selected biallelically mutated ESC clones and analyzed their phenotype in the founder generation as chimeric mice. Our approach provides a versatile tool for studying gene functions and generating disease models.

## Results

### sgRNA/CAS9-mediated NHEJ in ESCs

We previously showed that pronuclear injection of pX330 plasmids expressing sgRNAs and human codon optimized CAS9 efficiently generates pups with indels at the target sites (52.9 ± 22.3% of pups, n = 32 genes)^12^,^18^. In the present study, it was our goal to examine the feasibility of genome editing in ESC. We first transfected ESCs with pX330 plasmids which showed lower nuclease activity in fertilized mouse eggs (Fig. 2a, Table 1). The target sequences of sgRNA are shown in Supplementary Table S1. To concentrate the transfected cells, we co-transfected ESCs with pX330 plasmid and a pPGK-puro plasmid that expresses a puromycin resistant gene under the PGK promoter. We also used pX459, a modified pX330 that contains a puromycin-resistant gene expression cassette. The short period of puromycin treatment (2 to 3 days) was used to select for transiently transfected cells (only 8 out of 96 analyzed clones carried the integrated pX459 plasmids). In all ten targets, sgRNA/CAS9-mediated indels were frequently found in ESC clones (91.3%, Table 1). The absence of the wild-type (WT) allele in ESC clones confirmed that the biallelic mutations occurred (73.0% of clones analyzed), comparable to the previous report^10^.

Next, we examined the efficiency of sgRNA/CAS9-mediated excision between two cleavage sites (Fig. 2b, Table 2). In the fertilized eggs, the excision efficiency varied among the target genes and decreased as the distance was increased. In ESCs, the designed excision was obtained up to a distance of 841 kbp with 52.1% efficiency (Table 2).

### sgRNA/CAS9-mediated HDR in ESCs

sgRNA/CAS9-mediated DSBs and subsequent HDR with ssODN or dsDNA can be used to introduce designed mutations into the targeted locus^10^,^11^,^19^. To examine which reference target works better for small mutations (*e.g.*, point mutations and FLAG-tag insertion), we introduced sgRNA/CAS9 expressing plasmids with either ssODN or dsDNA references and examined HDR in fertilized eggs and ESCs (Fig. 2c, Table 3). We introduced mutations in the PAM sequence and/or adjacent 3′ seed sequence (8 nts) to prevent cleavage after HDR. The introduction of restriction sites enabled us to assess HDR without sequencing of PCR amplicons. We placed 50 ntsand 0.5 kbps homology arms into ssODN and dsDNA, respectively, for cost and practical reasons. With ssODN, targeted gene modifications were introduced into fertilized eggs (3.9% in 13 targets) and ESCs (2.7% in 7 genes) at similar frequencies (Table 3, Supplementary Table S2). With dsDNA, we failed to introduce the designed mutation into fertilized eggs (0% in 4 genes), but we obtained designed mutant ESCs at a high frequency (41.3% in 10 genes) (Table 3, Supplementary Table S3).

Because synthetic oligonucleotides are restricted to ~200 nts, we cannot use ssODN to knockin a larger fragment such as an EGFP reporter gene. Therefore, we constructed reference plasmids carrying the knockin cassette with either 0.5 kbps or 1.0 kbps homology arms (Fig. 2d, Table 4). When we injected fertilized eggs with the pX330 and the reference plasmids carrying 0.5 kbps homology arms, homologous recombination mediated EGFP knockin occurred at the *Centrin1* (*Cetn1*) locus in 4.0% of pups (2/50) (sgRNA #22 in Table 4 and Supplementary Table S1). With the same strategy, we transfected ESCs and confirmed that 15.0% of analyzed clones (18/120) were homologous recombinants. While the zeocin cassette was not knocked into the *Rosa26* locus in fertilized eggs (0/31 and 0/27 pups with 0.5 kbps and 1.0 kbps homology arms, respectively), correct knockin was observed in ESCs (10/225 and 18/224 clones with 0.5 kbps and 1.0 kbps homology arms, respectively) (sgRNA #42 in Table 4 and Supplementary Table S1).

### ESC-derived chimeric analysis in founder generation

Biallelic mutations are rarely obtained with conventional homologous recombination approaches in ESCs. However, as we showed here, biallelic mutations are efficiently obtained in ESCs using sgRNA/CAS9-mediated genome editing (Table 1). Therefore, we generated chimeric mice with these biallelic mutant ESCs to observe their phenotypes in the founder generation. To distinguish the mutant ESC-derived cells from WT host cells, we used ESC clones established from double transgenic mice expressing GFP ubiquitously and in the sperm acrosome^20^,^21^. In this mouse line, cells were labeled with EGFP in their cytosol and sperm cells were labeled with EGFP in their acrosome.

As a proof of concept, we first generated chimeric mice from ESCs carrying biallelic mutations in the *Cetn1* locus with sgRNA #22 (Fig. 3a, Supplementary Fig. S1, Supplementary Table S1). Observation of testes from chimeric mice under a fluorescent microscope showed the seminiferous tubules carrying green fluorescent spermatogenic cells (Fig. 3b). The malformation of sperm heads with defective cilia formation is consistent with previous studies of *Cetn1* knockout mice^12^,^22^ (Fig. 3c). It should be noted that we observed control WT cells in the same field enabling us to compare phenotypes without any biases.

Finally, we applied this method for a lethal gene, DnaJ heat shock protein family (Hsp40) member B13 (*Dnajb13*), which encodes one of the radial spoke proteins in the 9 + 2 structure^23^. As shown in Fig. 4, when we generated mutant mice by zygote injection of sgRNA #43 (Supplementary Table S1), homozygous mutant mice died before sexual maturation due to severe dilatation of the lateral ventricle and the abnormal increase of cerebrospinal fluid, indicating hydrocephalus (Fig. 4a,b, Supplementary Fig. S2a and S2b). However, most of the chimeric mice generated with biallelic mutant ESCs improved hydrocephalus symptoms and allowed survival until sexual maturation (Fig. 4c, Supplementary Fig. S2a and S2c). Interestingly we observed cilia in the ependymal cells of the lateral ventricles in *Dnajb13* KOs as well as in rescued mice (Supplementary Fig. S2b and S2c). Collection of the epididymal spermatozoa from the chimeric mice showed that the mutant cells were immotile due to sperm tail defects as observed in fertilization medium (Fig. 4d, Supplementary Movies S1–S4).

## Discussion

**Figure 1:**
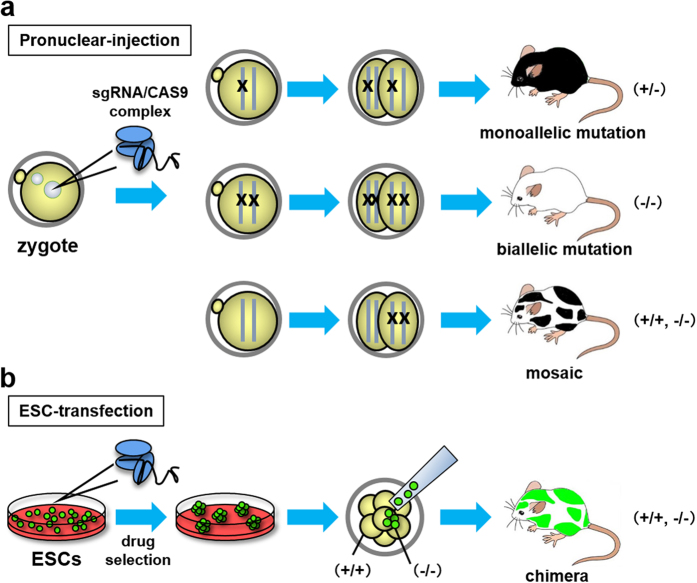
Experimental designs of the pronuclear-injection and the ESC-transfection. (**a**) The sgRNA/CAS9 expressing plasmid was injected into pronuclei of fertilized eggs. The induction of monoallelic or biallelic mutations at the one cell stage results in mutant mice carrying one or two different mutations in all cell types. On the other hand, the introduction of a mutation after the 2-cell stage results in mosaic mice in the founder generation. “X” indicates the mutated sites. (**b**) Mouse ESCs with GFP fluorescence were transfected with sgRNA/CAS9 expressing plasmids. The ESC clone with the desired mutation can be expanded and injected into WT (+/+) 8-cell embryos to generate chimeric mice. The ESC-derived mutant cells can be identified by fluorescence (green). (**a,b**) The mosaic mice in (**a**) may carry multiple cell types with unidentified mutations whereas chimeric mice in (**b**) carries WT (+/+) host cells and cells with identified mutations (−/−).

**Figure 2:**
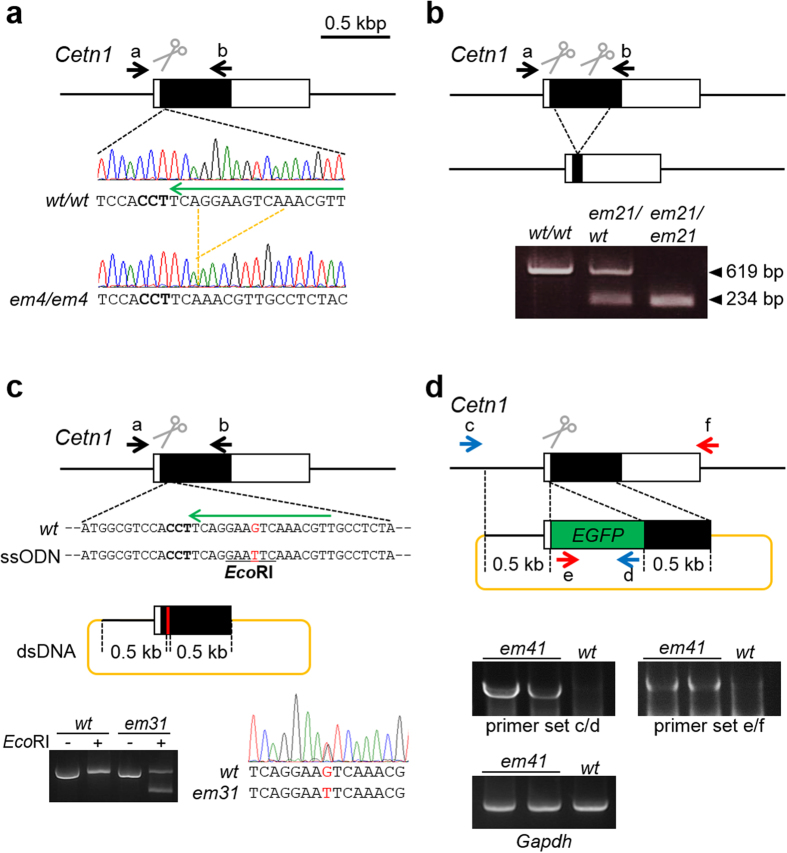
CRISPR/Cas9 mediated genome editing and screening strategy. (**a**) Indels introduced with a sgRNA/CAS9 expressing plasmid were identified by PCR using the primer set a/b (see Supplementary Table S1) and subsequent sequencing. The WT allele (*wt*) and the deletion mutant allele lacking 8 nts (*em4*) are presented. (**b**) Deletions introduced with two sgRNA/CAS9 expressing plasmids were identified by PCR using the primer set a/b and agarose gel electrophoresis. The WT and the mutant alleles lacking 385 nts (*em21*) have 619 bp and 234 bp, respectively. (**c**) Point mutations introduced with a sgRNA/CAS9 expressing plasmid and the oligonucleotide (ssODN) or plasmid (dsDNA) for the reference were identified by PCR using the primer set a/b and subsequent restriction enzyme digestion and/or sequencing. The ssODN and dsDNA contained 50 nts and 0.5 kbps homology arms, respectively. G to T mutation (*em31*, red-colored site) was introduced to generate *Eco*RI site (underlined sequence). PCR amplicons digested with (+) and without (−) *Eco*RI were subjected to electrophoresis and sequencing. (**d**) Gene knockins introduced with a sgRNA/CAS9 expressing plasmid and the reference plasmid (dsDNA) were identified by PCR. The homologous recombinant allele (*em41*) generates bands with primer sets c/d and e/f (see Supplementary Table S6). (**a–d**) The black box, scissors, bold fonts, and long green arrows indicate the coding region, DSB sites, PAM sequences, and sgRNA target, respectively. Primer pairs used for genotyping were indicated as short arrows (black, red, and blue).

In the present study, although it cannot be directly compared, we show that complicated genome editing is highly efficient in ESCs even with sgRNAs that do not work well in fertilized eggs (Tables 1, 2, 3 and 4, Supplementary Tables S2 and S3). There are additional advantages in ESC-mediated approaches. Whereas animals born without designed mutations are sacrificed after zygote injection, we can screen hundreds of ESC clones to obtain the ideal mutant clones without sacrificing animal lives. It should also be noted that researchers first believed that homozygous mutant mice could be obtained at the founder generation but later realized that mosaicism was highly prevalent in the founder generation which complicated genotyping and phenotyping analyses (Fig. 1)^16^,^17^. Moreover, there remains a risk of non-germ line transmission of the desired mutation because the germ cells and the cells used for genotyping might be different in mosaic animals after zygote injection. With ESCs, chimeric animals can be generated from clones carrying the identified mutations. If necessary, the desired mutation can be transmitted through the germline by crossing with WT animals.

**Figure 3:**
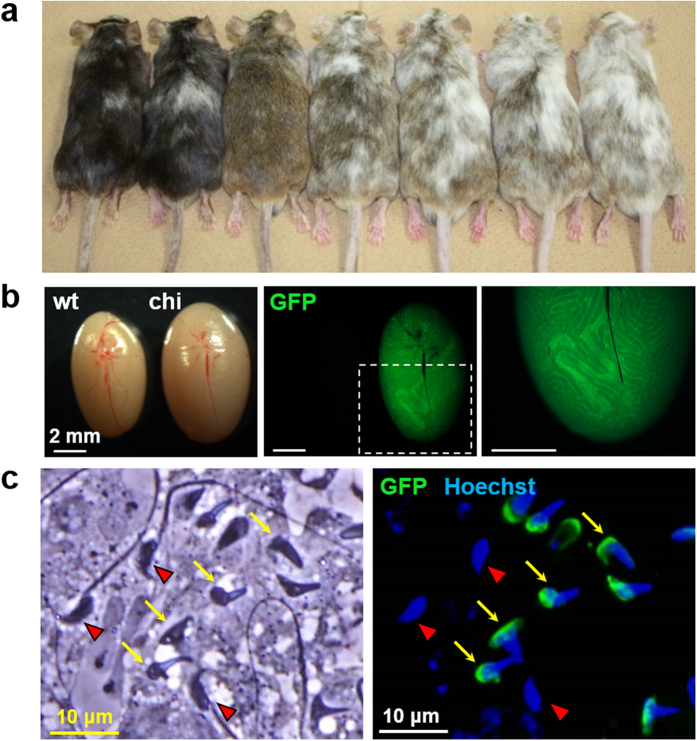
Functional analysis of *Cetn1* in the chimeric mice at founder generation. (**a**) Chimeric mice generated from ESCs carrying a biallelic mutation in *Cetn1* gene (*em51/em52*) (see Supplementary Fig. S1). Highly chimeric animals have darker coat color. (**b**) Testes of B6D2F1 (wt) and chimeric mice (chi) were photographed under a stereomicroscope (left panel; bright field, center and right panels; fluorescent field). The dashed-line square in the center panel was magnified in the right panel. (**c**) The cells dispersed from green fluorescent seminiferous tubules of a chimeric mouse were stained with Hoechst 33342 and photographed under a fluorescence microscope (left panel; bright field, right panel; fluorescent field). GFP-negative spermatozoa derived from host ICR and GFP-positive spermatozoa derived from mutated ESCs were indicated with red arrowheads and yellow arrows, respectively.

**Figure 4:**
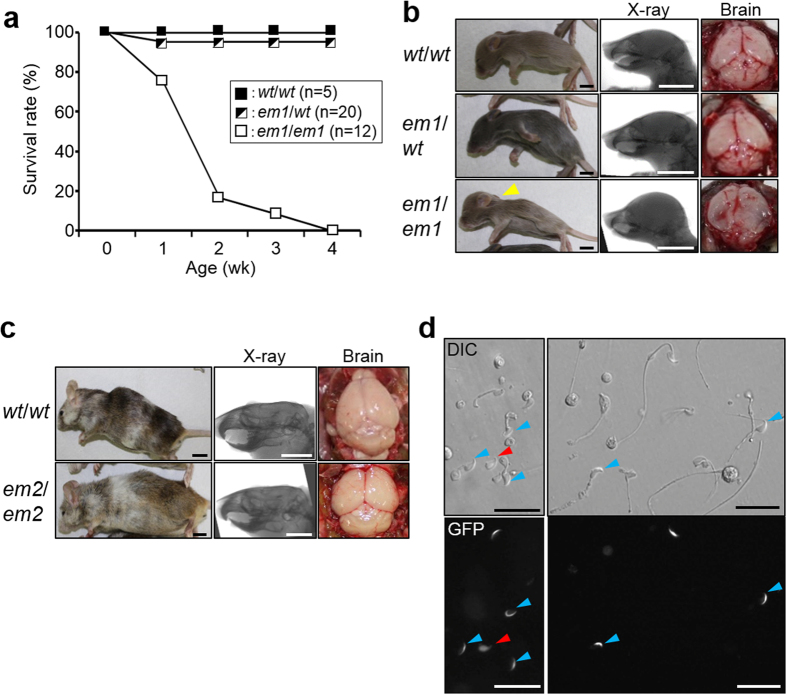
Sperm malformation found in chimeric *Dnajb13* KO mice rescued from hydrocephalus. (**a**) The survival rate of *Dnajb13* mutant mice (*em1/em1*) generated by pronuclear injection. (**b**) The brain morphology of 1 week old *Dnajb13* mutant mice (*em1/em1*) generated by pronuclear injection. A characteristic domed head (left panel, yellow arrowhead), the ventricular distention (center panel) and the abnormal accumulation of cerebrospinal fluid (right panel) were observed in KO mice (*em1*/*em1*) (see Supplementary Fig. S2b). Bar scales indicate 10 mm. (**c**) The brain morphology of adult chimeric mice generated with WT and KO ESCs (*wt/wt* and *em2/em2*, respectively). The chimeric mice survived to adulthood with mild hydrocephalus (see Supplementary Fig. S2c). Bar scales indicate 10 mm. (**d**) The DIC and fluorescent images of spermatozoa collected from the chimeric mice with *Dnajb13* KO ESCs (*em2*/*em2*). Red and blue arrowheads show tailless and short-tailed spermatozoa, respectively. These spermatozoa were immotile (see Supplementary Movies S1–S4). Bar scales indicate 25 μm.

If the founder mice obtained by zygote injection are mosaic, they can be crossed to obtain the heterozygous F1 generation and the subsequent F1 intercrosses can be used to obtain the homozygous F2 generation for phenotypic analysis (about two months for one generation in mice). Thus, the amount of time is similar to the conventional ESC mediated approach in which only one allele is mutated by homologous recombination or gene trap. In contrast, most ESC clones are biallelic mutant after sgRNA/CAS9-mediated genome editing, although the mechanism remains unclear. Therefore, one can analyze the effects of genome editing by differentiating the mutant ESCs *in vitro*^24^,^25^,^26^. Alternately, by using EGFP-tagged ESCs, one could directly analyze the mutant cell phenotypes in chimeric mice at the founder generation (Figs 3 and 4, Supplementary Movies S1–S4).

Our approach also gives us a chance to analyze lethal genes *in vivo* without a Cre-mediated conditional knockout (cKO) strategy (Fig. 4). We chose *Dnajb13* because KO mice die due to hydrocephalus. As we expected from previous studies showing abundant testis expression^23^,^27^, the radial spoke protein DNAJB13 is required for functional sperm formation (Fig. 4d and Supplementary Movies S1–S4). In contrast, *Dnajb13* KO cilia looked normal in the lateral ventricle (Supplementary Fig. S2b), implicating that the DNAJB13 plays a more critical role in sperm flagella formation than ciliary formation. This is consistent with previous data found in KO mice for cilia and flagella-associated proteins, CFAP221^28^ and CFAP54^29^. Different from a cKO approach, all the ESC-derived cells in the body carry KO mutations. Thus, the chimeric mice can also be used as a source of mutant cells, enabling us to isolate, expand, and analyze their fate. In combination with stem cell transfer technology^30^,^31^, whole systems like the hematopoietic cells can be reconstituted in recipient animals which would accelerate gene functional studies *in vivo*. In many cases, analysis can be achieved without further time consuming and laborious breeding steps.

Recently, next generation sequencing has identified numerous genetic variations among human populations^32^,^33^. To validate if the variation is associated with a particular disease, creation of the same mutations in mice is a critical step. Different from null mutations that can be caused by simple DSB and subsequent NHEJ, these genetic variations need to be introduced precisely by HDR. We show here that dsDNA rather than ssODN works well with CRISPR/Cas9 in ESCs while no such success was observed by zygote injection. Further, some researchers require multiple mutations in one individual to characterize diseases caused by multiple factors. These multiple mutations can be efficiently introduced with the CRISPR/Cas9 system^10^,^34^,^35^. We conclude here that the combination of genome editing in ESCs and subsequent chimeric analysis provides a robust tool not only for reverse genetics but also for genome-wide association studies.

## Methods

### Animals

All animal experiments were conducted in accordance with the guidelines of “Animal experiment rules” established by the Research Institute for Microbial Diseases, Osaka University, and were approved by the

Animal Care and Use Committee of the Research Institute for Microbial Diseases, Osaka University. B6D2F1 and ICR mice were purchased from CLEA (Tokyo, Japan) and SLC (Shizuoka, Japan).

### Plasmid construction and genotyping

The construction of sgRNA/CAS9 expressing plasmids, pX330 and pX459 (#42230 and #48139, respectively, Addgene, Cambridge, MA, USA), and the validation of the DNA cleavage activity of these plasmids were performed as described previously^12^,^36^. The sgRNA oligonucleotides and genotyping primers are listed in Supplementary Table S1. Genotyping primers were located outside of the homology arm region to distinguish from the transgene that integrated into the genome. The reference oligonucleotides used for HDR are listed in Supplementary Table S4. The reference plasmids used for HDR were constructed in pBluescript II SK (+) vector containing EGFP or the zeocin cassette. Homology arms were amplified by PCR with primer sets shown in Supplementary Table S5 and placed in 5′ and 3′ sites of the EGFP or zeocin cassette. HDR was screened by PCR with primers listed in Supplementary Table S6.

### Pronuclear injection of mouse zygotes

B6D2F1 superovulated females were mated with B6D2F1 males and then fertilized eggs were collected. Circular pX330 plasmids were injected into one of the pronuclei at 5 ng/μl with or without reference DNA (ssODN at 100 ng/μl and dsDNA at 100 ng/μl)^12^,^36^.

### Genome editing in mouse ESCs and generation of chimeric mice

The EGR-G101 ESC line was previously established from C57BL/6-Tg (CAG/Acr-EGFP) C3-N01-FJ002Osb^21^. The 1 × 10^3–4^ EGR-G101 ESCs were seeded on mouse embryonic fibroblasts (MEF) in a 6 well plate and transfected with sgRNA/CAS9 expressing plasmids (total 1.0 μg) using Lipofectamine LTX & PLUS technology (Life Technologies, Carlsbad, CA, USA). When the pX330 plasmids were used, a pPGK-puro plasmid (0.1 μg) was also transfected. For HDR experiments, the ESCs were co-transfected with 1.0 μg of ssODN or dsDNA. After 14–18 hours of transfection, the cells were selected with puromycin (0.1 μg/ml) for 48 hours, then grown for 5 to 6 more days, picked, and transferred onto MEF cells in 96-well plates. After 48–72 hours of culture, each ESC clone was split in duplicate, for freezing, and DNA harvesting. After PCR amplifications and direct sequencing, the positive clones were thawed and expanded to analyze their karyotypes. The mutant ESC clones were injected into 8-cell ICR embryos, and the chimeric blastocysts were transported into the uteri of pseudopregnant females^37^.

### Sperm motility and morphology of chimeric mice

The cauda epididymal spermatozoa were collected from chimeric mice. Some spermatozoa were incubated in modified Krebs–Ringer bicarbonate (TYH) medium for 10 min and then the sperm motility was observed. To observe the morphology, spermatozoa were dispersed in PBS and then stained with 65 μM Hoechst 33342 (Life Technologies) for 5 min.

### Brain morphology

The X-ray images of brains were taken under anesthesia (Isoflurane: Mylan Inc., Canonsburg, PA, USA) using *In-Vivo* Multispectral Imaging System FX (Kodak, New Haven, CT, USA). The brains of 1-week old mice were fixed in 4% PFA for 2~7 days, dehydrated by EtOH and xylene, and then embedded in paraffin. The brains of adult mice were fixed in 1~2 days, dehydrated by 100% acetone, and subsequently embedded in plastic resin (Technovit 8100, Heraeus Kulzer GmbH, Hanau, Germany). The 5-μm sections were subjected to hematoxylin and eosin staining and then enclosed with Entellan New (Merck, Darmstadt, Germany).

## Additional Information

**How to cite this article**: Oji, A. *et al.* CRISPR/Cas9 mediated genome editing in ES cells and its application for chimeric analysis in mice. *Sci. Rep.* **6**, 31666; doi: 10.1038/srep31666 (2016).

## Supplementary Material

Supplementary Information

Supplementary Movie S1

Supplementary Movie S2

Supplementary Movie S3

Supplementary Movie S4

## Figures and Tables

**Table 1:** Efficiency of generating indel mutant mice or ES clones via pX330 plasmid injection or transfection.

sgRNA	Chr.	Pronuclear-injection						ESC-transfection					
		Analyzed pups	Mutation					Analyzed clones	Mutation			GM efficiency^a^(%)	
			Single	Double	Mosaic	GM efficiency^a^ (%)			Single	Double	Mosaic		
#01	9	41	0	0	2	2	(4.9)	19	0	17	1	18	(94.7)
#02	11	14	0	0	0	0	(0)	13	0	12	0	12	(92.3)
#03	4	22	1	0	0	1	(4.5)	25	1	10	10	21	(84.0)
#04	16	17	0	0	0	0	(0)	13	1	10	2	13	(100)
#05	17	21	0	0	0	0	(0)	8	1	7	0	8	(100)
#06	6	19	1	0	1	2	(10.5)	8	0	8	0	8	(100)
#07	7	17	2	0	0	2	(11.8)	8	2	4	1	7	(87.5)
#08	18	13	0	0	0	0	(0)	7	0	7	0	7	(100)
#09	18	11	0	0	0	0	(0)	6	0	5	0	5	(83.3)
#10	7	12	0	0	0	0	(0)	8	1	4	1	6	(75.0)
total	—	187	4	0	3	7	(3.7)	115	6	84	15	105	(91.3)

Chr.: chromosome number, Single: monoallelic mutant, Double: biallelic mutant, GM: Genetically Modified.

^a^Total of mutations/analyzed pups or clones.

**Table 2:** Efficiency of generating deletion mutants by introducing two sgRNAs into zygote or ES cells.

sgRNAs	Chr.	Length (kbp)	Pronuclear-injection			ESC-transfection		
			Analyzed pups	Deletion (%)		Analyzed clones	Deletion (%)	
#11 + #12	5	0.4	10	2	(20.0)	24	11	(45.8)
#13 + #14	7	20	6	1	(16.7)	16	12	(75.0)
#15 + #16	6	60	13	3	(23.1)	32	7	(21.9)
#17^a^	16	130	21	0	(0)	96	5	(5.2)
#18 + #19	Y	380	—	—	—	32	6	(18.8)
#20 + #21	9	841	13	0	(0)	48	25	(52.1)

Chr.: chromosome number.

^a^A sgRNA recognizes the same sequence with 130 kbp apart.

**Table 3:** HDR-mediated small mutations using ssODN or dsDNA as a reference.

Reference	Pronuclear-injection					ESC-transfection			
	Number of targets^a^	Analyzed pups	GMO	HDR (%)		Number of targets^a^	Analyzed clones	HDR (%)	
ssODN	13^b^	232	58	9	(3.9)	7^c^	448	12	(2.7)
dsDNA	4^d^	48	8	0	(0)	10^e^	247	102	(41.3)

GMO: Genetically Modified Organism, HDR: Homology-Dependent Repair.

^a^The sgRNAs used for this experiment are shown in Table S1, and the breakdown of this table is shown in Tables S2 and S3.

^b^sgRNAs #22 ~ #34,

^c^sgRNAs #22 ~ #24, #29, #30, #35, #36,

^d^sgRNAs #22 ~ #24, #27,

^e^sgRNA #22 ~ #24, #35 ~ #41.

**Table 4:** HDR-mediated reporter knockins using dsDNA as a reference.

sgRNA	Chr.	Homology arms (kbp)	Pronuclear-injection				ESC-transfection		
			Analyzed pups	GMO	HDR (%)		Analyzed clones	HDR (%)	
#22	18	0.5 + 0.5	50	5	2	(4.0)	120	18	(15.0)
#42	6	0.5 + 0.5	31	12	0	(0)	225	10	(4.4)
		1.0 + 1.0	27	0	0	(0)	224	18	(8.0)

GMO: Genetically Modified Organism, HDR: Homology-Dependent Repair.
