# Supplementary material for: CRISPR/Cas9 mediated genome editing in ES cells and its application for chimeric analysis in mice
Source: Sci Rep. 2016 Aug 17;6:31666. doi: 10.1038/srep31666 (PMC4987700; doi:10.1038/srep31666)
Supplement: Supplementary Information [file srep31666-s1.pdf]

Supplementary Information

Title

CRISPR/Cas9 mediated genome editing in ES cells and its application for chimeric analysis in mice

Authors

Asami Oji, Taichi Noda, Yoshitaka Fujihara, Haruhiko Miyata, Yeon Joo Kim, Masanaga Muto, Kaori Nozawa, Takafumi Matsumura, Ayako Isotani, Masahito Ikawa*

Corresponding author

Masahito Ikawa, Ph.D.,
Research Institute for Microbial Diseases, Osaka University,
3-1 Yamada-oka, Suita, Osaka 5650871 JAPAN
Phone: +81-6-6879-8375, Fax: +81-6-6879-8376
E-mail: ikawa@biken.osaka-u.ac.jp

Contents

Supplementary Figures S1-S2
Supplementary Tables S1-S6
Supplementary Movies S1-S4

Supplementary Figure Legends

Supplementary Figure S1. Generation of *Cetn1* mutant ESCs
(a) *Cetn1* was targeted in ESCs with sgRNA #22 (see **Supplementary Table S1**). The black box, scissors, and arrows indicate the coding region, DSB site, and primer set a/b (see **Supplementary Table S1**), respectively.
(b) Biallelic deletions were identified by sequencing. The 330 bp and 268 bp were deleted between black and red character in *em51* and *em52*, respectively. Green arrow indicates the sgRNA target.

Supplementary Figure S2. Suppression of hydrocephalus and lethality in *Dnajb13* chimeric KO mice
(a) *Dnajb13* exon 8 was targeted in ESCs with sgRNA #43 (see **Supplementary Table S1**). The black boxes, scissors, and arrows indicate the coding region, DSB site, and primer set g/h (see **Supplementary Table S1**), respectively.
(b) *Dnajb13* mutant mice (*em1*/*em1*) generated by pronuclear injection. The deletion of 2 bp was identified by PCR and sequencing (i). The brain sections were prepared from 1 week old mice (upper panels) and observed after HE staining (lower panels) (ii).
(c) Chimeric mutant mice generated with WT and KO ESCs (*wt*/*wt* and *em2*/*em2*, respectively). The deletion of 90 bp was identified by PCR and sequencing (i). The brain sections were prepared from adult mice (upper panels) and observed after HE staining (lower panels) (ii). (b and c) Cb, Hp and LV indicate the cerebellum, hippocampus and lateral ventricle, respectively. A region of the LV shown as dashed-lined squares was magnified and then cilia were observed (arrowheads).

Supplementary Figure S1

a

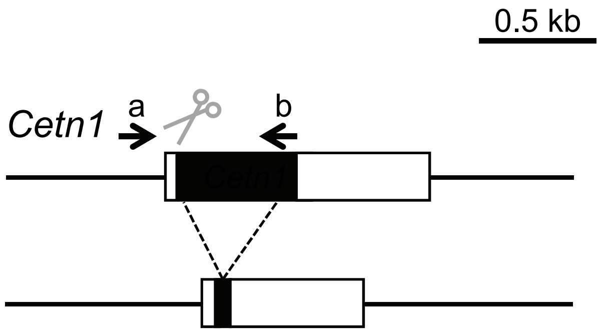

b

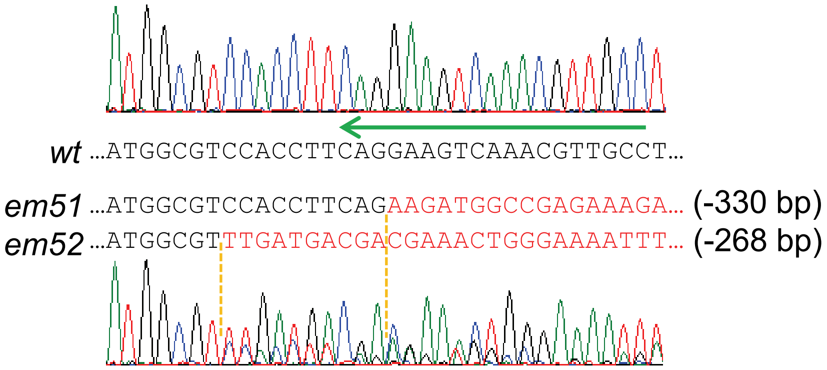

Supplementary Figure S2

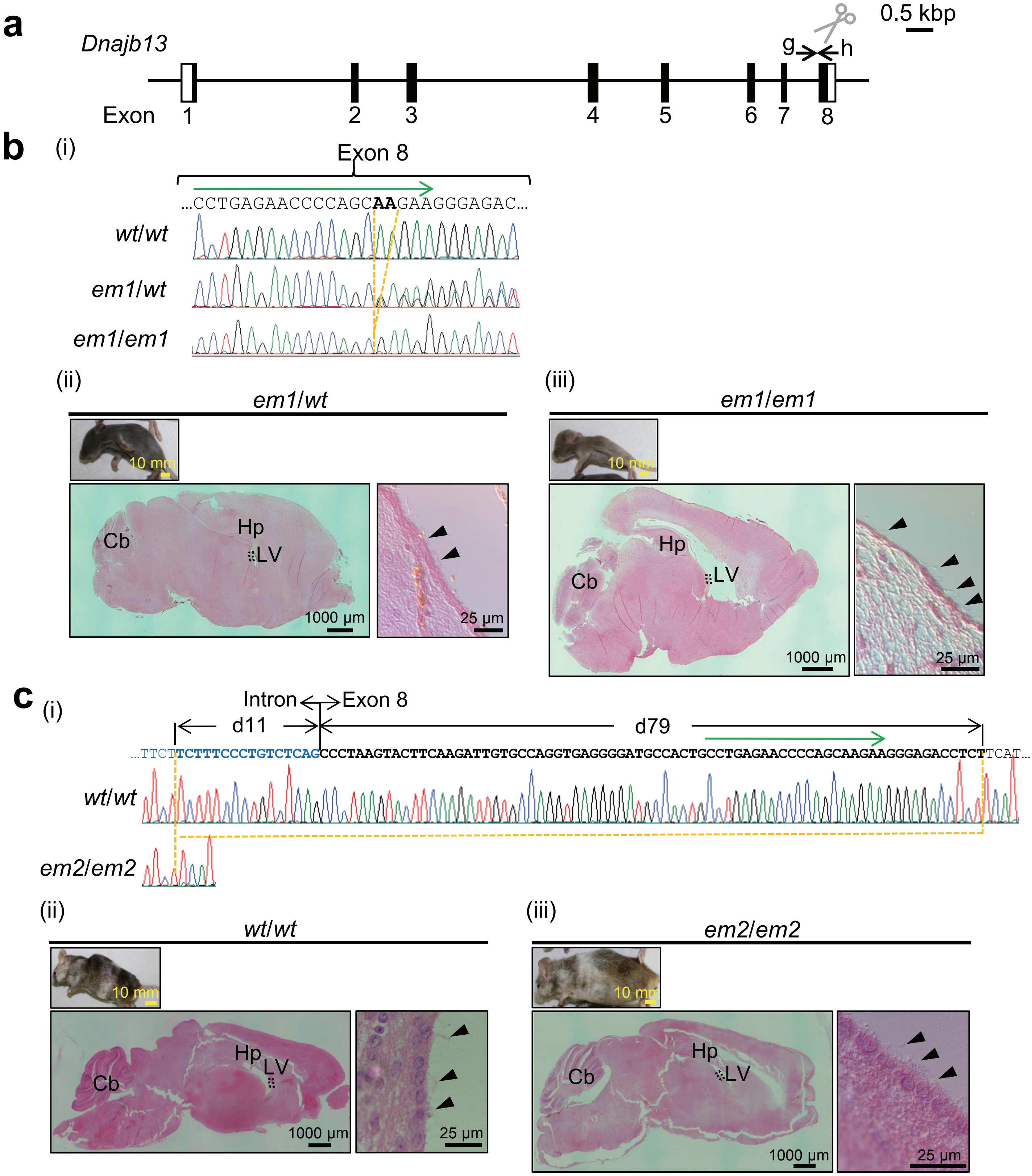

Supplementary Table S1. Target sequence of sgRNAs and oligonucleotides used for genotyping

sgRNA	target sequence (5' to 3')	PAM		PCR primer sequence (5' to 3')
#01	GAGGTTCTTGCTTGTGGCGC	TGG	F<br>R	CCCCTGTTGGTCAGTTTGCTTTCTACATTC<br>GGAATGTTCCTCCTCCCACAGCC
#02	GGCCAAGGTTGGGCTACTCC	AGG	F<br>R	GGGTATGTCTTTCTCTCAGGGTCCTAGAG<br>GCCTTGGCTTCATCAGTCTCTAGGT
#03	TTCCAGCAAGATGTAAGCTT	TGG	F<br>R	GGTGGAAGCTGAGACAGCCC<br>ATAAACACGGACTAACCTGC
#04	CCCCAGTGAGGGTCCGCACC	AGG	F<br>R	GGGCAGGGTGGGAACAATCAAT<br>GCAGCCTGTAGAAAGGAAAGTCGAGG
#05	GAGCAACAGGAGGTGTTTCA	TGG	F<br>R	GGCCCTCAGCTGGGGGTCC<br>CCAGCATTAGAAGGTAGAGGCAGGAGG
#06	TCTGATAGTGAGTGCAAGCT	TGG	F<br>R	CCTTCTGTCTGGCAATGGAAT<br>TGACAACTGTAGGTAGCCCCA
#07	GTTAGAGTGATGCACAAGGTT	TGG	F<br>R	CACGTGTGATGCCACAGGTGCC<br>GCAAGTAAGCCTCTGGGCTGTCC
#08	CAAAAGATGGCGACGCCCCT	TGG	F<br>R	ATTGAACCACAAGCCCGGCTCT<br>TGAGGTCTCTACCGCTAAACGTGGA
#09	GGGTGGTCGCAGGGGGGCTC	TGG	F<br>R	ATTGAACCACAAGCCCGGCTCT<br>TGAGGTCTCTACCGCTAAACGTGGA
#10	CAGAACACACAAATGGCGGA	AGG	F<br>R	GGGGAGAACAACCAATTGTCAGCCC<br>CTTGAAGCCATTGGGCCAGTTTGTTTG
#11<br>#12	CCACAAGGAACTGAACGACC<br>ACAACAGAACAGCTCGGGAA	TGG<br>TGG	F<br>R	AGGCCAGAACTGCCTGCCTC<br>CATGGGAAACAGTCCGGTGG
#13<br>#14	CAGGAAATATGGCACGAGCT<br>GGTGATCGAGAGATAATACG	AGG<br>CGG	F<br>R	TACTTAGTCCACTCTGTACC<br>GGTCAGCGTTACAGTGAAGG
#15<br>#16	CATCACAGTAAACAGTCCAA<br>CTTCTGCGAGCTCATGAAGT	AGG<br>TGG	F<br>R	GACCACATCTTTCATGTCC<br>AGCAACTGAGAATGCAACCC
#17	CATCTATCTCATCACCATTG	TGG	F<br>R	GTTTCATTTCAGGGACACCC<br>CCACACACACAGACCATTGG
#18<br>#19	ATCATGACAATCACAACTCG<br>TGAGGAAAAGAAAATGGCGG	TGG<br>CGG	F<br>R	AAATTAGAGATTTTAGGGCCTGGCATATGG<br>TTTCAGAGTAGGGAGAGGCTCCAG
#20<br>#21	AGGTATTTTCCAATTGCAGT<br>TTGTACAAGTCTCATTCAAT	GGG<br>GGG	F<br>R	CCCTTGAACCTGGGATTGTTT<br>AGCCAGCCTCTAGTGGAGACA
#22	GGCAACGTTTGACTTCCTGA	AGG	F (a)<br>R (b)	TGCAGGCGCATAGGGGCTGG<br>AGTCACTTAATAAAGGTTGG
#23	AGCCACACATTTGATCTTCA	GGG	F<br>R	CTTTGAAGACCCAGGAAAGG<br>AAATGTCCTTAAGGAGTCCC
#24	CATCACAGTAAACAGTCCAA	AGG	F<br>R	TAAGACAGAGGGGAAAGATTGG<br>AGGGGCTAATTGCTCTCTTGG
#25	CACTTGATCAAACATTACCC	AGG	F<br>R	TGGAGTCTGAGCAGATCCTG<br>ACAGCGATATCCCTAACCCC
#26	GTGTTAGAGGATGAGCTAAC	AGG	F<br>R	ATAAACCTTGGACATGGTAG<br>TGGGACTTTACAAAGCAAG
#27	GCTGTCTGGAAATTGACTGC	TGG	F<br>R	ATGACTAGGGAGGAGCAGAGAC<br>ATTCCCATGACCACTCACTACC
#28	TACTCTGGTGATGAATGCCG	CGG	F<br>R	GAAAGATGCTGCGCCAGAGG<br>TAGCATCAAGGAGAATCTGG
#29	GAGAGATACTCAGAAGCTGCC	CGG	F<br>R	CTACGTCAGTGCTGCCAAGTAGC<br>CCATCATCACTAATGACCTCATGGCTGG
#30	GTCACATCCAGGACCCTGACA	CGG	F<br>R	GGAGAACAGCCTGTGTAATGGTGGG<br>CAAGACAGGGTTTCTCTGTGCTCCC
#31	GTTCCTAAAGTGAAGTACCCT	GGG	F<br>R	GGATGGATGGAAAGGAAACG<br>GGAGCCAGAAGTCAGAACCA
#32	GAGAAAGGAAGGCCAGATCA	CGG	F<br>R	ACTTCGTCTATTCTGCTCCGG<br>ATGAGTCAGAGTGTGCACAGC
#33	AACAAGCGTCCAAGATGCTG	AGG	F<br>R	AGGTCGATTGTCTGTGTGG<br>CTCATTCCCAAGATGCTAGG

#34	GTTCGAGGAAATCATGAATGC	AGG	F<br>R	GGTAGCTTTGCCTAGAGAGTCC<br>ACCTTGTCAAATGGAGACTACC
#35	CGGCCCAGATCATGAAATCA	TGG	F<br>R	CATTGTGATGAAAGACTTGAGGG<br>ATTCTCTCCCCGACCTGACC
#36	TTTTGTTGATGACTTTCTGT	TGG	F<br>R	TCCATCACCACCACCAGGTG<br>TCTGTGCCTCAGGCCTTGGC
#37	GTCCCCGGTTCCTGGAGCAAC	TGG	F<br>R	AGAGAGAGATGGCTTAGCCG<br>GCAAAACACCCAAACACATC
#38	GTCATGAGCAAAGTCATCAGT	AGG	F<br>R	TGTTCTAGTTAGTGCGTTGG<br>CACCCACGTCATGAGAGAAG
#39	GAGACAGGGTTAGTCCAAAA	TGG	F<br>R	CAGTGAGTGCCTAGGTGAAC<br>CTCAGCTGCTGCTCCAGTAG
#40	GAATTCTGTAGTCTAAGATG	TGG	F<br>R	CTAGACCGACTATTCTCAGG<br>TGTACAAGCAGACAACGTCAGG
#41	AGGGATAGACGGTGATCTGA	TGG	F<br>R	CTAGACCGACTATTCTCAGG<br>TGTACAAGCAGACAACGTCAGG
#42	GTTGCAGATCACGAGGGAAG	AGG	F<br>R	GCTGCGGGGCGGGTGC<br>CTGCTTACATAGTCTAACTCGCGACACTG
#43	CCTGAGAACCCCAGCAAGAA	GGG	F (g)<br>R (h)	GCTCTGGTGTGTGAGACCTG<br>TAGAGATAGTCTGTTGTGAG

Supplementary Table S2. HDR-mediated small mutations using ssODN as a reference

pronuclear-injection					ESC-transfection			
sgRNA	analyzed pups	GMO	HDR (%)		sgRNA	analyzed clones	HDR (%)	
#22	13	5	1	(7.7)	#22	56	7	(12.5)
#23	29	17	0	(0)	#23	24	0	(0)
#24	5	0	0	(0)	#24	16	2	(12.5)
#25	8	5	2	(25.0)	-	-	-	-
#26	4	1	1	(25.0)	-	-	-	-
#27	6	2	0	(0)	-	-	-	-
#28	26	0	0	(0)	-	-	-	-
#29	35	1	0	(0)	#29	96	0	(0)
#30	34	2	1	(2.9)	#30	96	0	(0)
#31	13	1	0	(0)	-	-	-	-
#32	27	9	1	(3.7)	-	-	-	-
#33	14	7	2	(14.3)	-	-	-	-
#34	18	8	1	(5.6)	-	-	-	-
-	-	-	-	-	#35	16	3	(18.8)
-	-	-	-	-	#36	144	0	(0)
total (13)[a]	232	58	9	(3.9)	total (7)[a]	448	12	(2.7)

GMO: Genetically Modified Organism, HDR: Homology-Dependent Repair.

[a]The numbers in parentheses indicate the number of targets.

Supplementary Table S3. HDR-mediated small mutations using dsDNA as a reference

pronuclear-injection					ESC-transfection			
sgRNA	analyzed pups	GMO	HDR (%)		sgRNA	analyzed clones	HDR (%)	
#22	25	2	0	(0)	#22	55	13	(23.6)
#23	15	3	0	(0)	#23	56	24	(42.9)
#24	2	0	0	(0)	#24	16	6	(37.5)
-	-	-	-	-	#35	16	13	(81.3)
-	-	-	-	-	#36	16	7	(43.8)
#37	6	3	0	(0)	#37	16	6	(37.5)
-	-	-	-	-	#38	16	5	(31.3)
-	-	-	-	-	#39	24	6	(25.0)
-	-	-	-	-	#40	16	7	(43.8)
-	-	-	-	-	#41	16	15	(93.8)
total (4) [a]	48	8	0	(0)	total (10) [a]	247	102	(41.3)

GMO: Genetically Modified Organism, HDR: Homology-Dependent Repair.

[a]The numbers in parentheses indicate the number of targets.

Supplementary Table S4. Oligonucleotides used for HDR-mediated point mutations or tag insertions

sgRNA-mutation	sequence (5' to 3')
#22-EcoRI	CAGGCTTAGGACCCACCTTTCTCTTGTAGCTGGTAGAGGCAACGTTTGAA TTCCTGAAGGTGGACGCCATCCTCCACAACAGTTGGGATCTTAACGGTGC
#23-FLAG	ACGTTTGGGATTTTTTCCTTTATATAATTAGCCACACATTTGATCTTCAG TGTTTTATCGTCGTCATCCTTATAATCCATGGCGGCAACGGGGCAGGAAC AGCTGAAGGAAGGGCATGGGACCTGCCATCCATTTT
#24-FLAG	TGCGAACCTTGAACCTTCCTCACTGTTTATTTATGTTTGGTACTTCAGAG AGGATTACAAGGATGACGATGACAAGTGACAGTCCAAAGGAATGTTGGTA CATCTTTTATTAACCATAAAATAAAAGACT
#25-PM	GAACGCGGCTCAGGTGATGGTAACACAACAGTCCCTCTTGGAGCACTTGA TTAAACATCATCCAGGTAAGCAATTTCCGCCCTTGGTGTTCATCCGAATG TGTTTGGTTTCC
#26-PM	CTTCACGTGGTCGTTTGGAACACCGTAGACTTCTATGATCACTACTATGT CAGGCGTGTTAGACAAGCTTGAAACAGGCAACTGGATCCCACTGATGATC TGTGCAAACACACATGACCAGAAGAACTTA
#27-PM	TATGACACTATTTTGCCTCCCTTTTGCTAGTGCTCTTGTGAAAGGCTGCC GCTGTCTGGAAATACGTTGCTGGGATGGATCCCAAAATGAACCCATTGTG TACCATGGTTACACATTCACCAGCAAGCTT
#28-PM	TGACTACAAATGTTTCAAACATTTTCATTTCTTTTTTCTCTTTGTAGTCC ATGAGTTTATTTTCCCCACAGCTGCATTCATCACCAGAGTATACCCCAAA ATGATGAGAGCAGACTCTTCTAACTTTAAC
#29-PM	AAACCATCTTCGTCAGGCAGCCGTGTTTAGATGTCTGGAGAGATACTCAG AGGCCGCCCAGTATGTTTGTTACAACTTTCTGAAGATTTATTTAAAGTTA AATTAATTTT
#30-FLAG	ACTGGCTCCAGAGTGACGTGTCACATCCAGGATCCTGACACGCCACCATG GACTACAAGGATGACGATGACAAGATACTGCAGGCCTGGAGATCTCTGCA GCTGCTGTACCTCTTAGAAGCCAT
#31-PM	TGATCTGACTTGGATCCTGACGCGGTGGGTTTGACTTCGTCACTTGCATT CTTCAATTTAGCGCTAGACTTTCCTAAAGTGAAGTACCCTGGGGCAGGAC ATAGGTGCACAGAACCACATTAGGTCACAG
#32-FLAG	GGCACAGGGTACTTGGCAGGTGGGAGAAAGGAAGGCCAGATCACGGAGCC TGATCATTTATCGTCGTCATCCTTATAATCTGGAGCTGGAGCCTCTGGGA AGACATGGCTTGCGTGATATGTTGAGCCCT
#33-FLAG	GGAGCAAGAGGTGGCCCTGTGCAGGGAAGGAGGAGCGGGAACAAGCGGCC GCCATGGATTATAAGGATGACGACGATAAACTGAGGGTTGTTGTGGAGTC CGCTTCGATTAACCCTCCCCTCAGCACCAC
#34-PM	GTGCTGTATCTATGGAGCTTAAAGATTAACCATCCTAAAACATTGTTTCT GCTTCGAGGAAATGCTGAATGCAGGCATCTTACAGAGTACTTCACCTTCA AACAGGAATGTGAGTATACATCTCTCCAGA
#36-PM	TCTGTGAGACCTCTTGATGTGTCTTTTATTTAGGTTGCTTTTACGTTTTG TGGCCGCCTTCCTGCTGGTGACGCCTCACTTGGACCAAGCAAAAACCTTC CTCAGGTAAGGACCATCAT

PM: point mutation.
Red-colored letters indicate point mutations and/or FLAG sequences.

Supplementary Table S5. Oligonucleotides used for cloning of homology arms

sgRNA	homology arm (kb)		sequence (5' to 3')
#22	5' (0.5)	F	CTGCCAGGAAAGTTTACTAAGG
#22	5' (0.5)	R	TTgaattcCTGAAGGTGGACGCCATCC
#22	3' (0.5)	F	AGgaattcAAACGTTGCCTCTACC
#22	3' (0.5)	R	AActcgagCAGTCACTTAATAAAGGTTGG
#23	5' (0.5)	F	TTggatccGGGTCAGGAAACTGTAAAGG
#23	5' (0.5)	R	TTgaattcCCATGGCGGCAACGGGGCAGGAACAGCTGAAGG
#23	3' (0.5)	F	CGCCATGGATTATAAGGATGACGACGATAAAACACTGAAG<br>ATCAAATGTGTGG
#23	3' (0.5)	R	TTctcgagCTCATTATCCTGCATGAGTTTGG
#24	5' (0.5)	F	TTctagaTGGATCATTTTCGGGGCTGG
#24	5' (0.5)	R	TTggatccTCACTTGTCATCGTCATCCTTGTAATCCTCTC<br>TGAAGTACCAAACAT
#24	3' (0.5)	F	TTggatccAGGAATGTTGGTACATCTT
#24	3' (0.5)	R	TTctcgagATAAGAGGCCACAAAAGGCC
#35	5' (0.5)	F	TTctagaTGAGAAAGAGTATGGTTCCC
#35	5' (0.5)	R	TTggatccCTTGTCATCGTCATCCTTGTAATCGATCATGA<br>TTTCATGATCTGGGCCG
#35	3' (0.5)	F	TTggatccGAAAGCCAACTTCATGAGCT
#35	3' (0.5)	R	TTctcgagTACAGAAGTCAGAGCACTGG
#36	5' (0.5)	F	TCTGTGAGACCTCTTGATGTGTCTTTTATTTAGGTTGCTT<br>TTACGTTTTGTggccGCCTTCCTGCTGGTGACGCCTCACT<br>TGGACCAAGCAAAAACCTTCCTCAGGTAAGGACCATCAT
#36	5' (0.5)	R	AAgaattcGGCAAATAGCCCTTCTGTGG
#36	3' (0.5)	F	AAtctagaGTGCTATACGCAGTGCCAGG
#36	3' (0.5)	R	CCACAAAACGTAAAAGCAACC
#37	5' (0.5)	F	AAggatccACCACATGGTGGCTCACAGC
#37	5' (0.5)	R	AAgaattcAATCCATGGCGGCCGTGCTGCCTTGTGGCTAGG
#37	3' (0.5)	F	CGccatggATTATAAGGATGACGACGATAAAGGCAAGACC<br>ATTCCCCGGTTCC
#37	3' (0.5)	R	AActcgagACTCTGGGCAAAAGGTGTCTC
#38	5' (0.5)	F	AAggatccGTGTCAGGAGACTAAAAGAGC
#38	5' (0.5)	R	AAgaattcATCCATGGCGGCTGGAGAAAGGAGAACGAC
#38	3' (0.5)	F	CGccatggATTATAAGGATGACGACGATAAAGGTACTAGG<br>AAAAAGGTTCAAGCG
#38	3' (0.5)	R	AActcgagCTGCAAGCTGCTCTCTGACTTCC
#39	5' (0.5)	F	ATtctagaCCATAGCTTCCTTCTCCTCC
#39	5' (0.5)	R	TGgatatcTTAGGTGCTGTCCAGGCCCAGCAGGGGGTTGG<br>GGATGGGCTTGCCGTCCAAAATGGTCTGAAAGTCTGTGC
#39	3' (0.5)	F	AAgatatcCATTGTAGCCTCAGCACTCC
#39	3' (0.5)	R	AActcgagAAAATCCTGCTTTTATAAGTTGG
#40	5' (0.5)	F	AAgctagcACCCAAGACTGAAATGAAGG
#40	5' (0.5)	R	AAgaattcTCAGTACAGTTGTTAGTGG
#40	3' (0.5)	F	GAgaattcTGTAGTCGACTACAAAGACGATGACGACAAGT<br>AAGATGGGTTACCCCGGCAACC
#40	3' (0.5)	R	AActcgagATCTCCAGGGAGGGTGGGAGG
#42	5' (0.5)	F	AAgagctcGGTCAGCGAAAGTAGCTCGC
#42	5' (0.5)	R	AAccgcggAAAGGTATTGCAACACTCCC
#42	3' (0.5)	F	AActcgagGTGGGCGTTGTCCTGCAGGG
#42	3' (0.5)	R	TTggtaccATGCCAATGCTCTGTCTAGG
#42	5' (1.0)	F	AAgagctcAGGCCCTCCGAGCGTGGTGG
#42	5' (1.0)	R	AAccgcggAAAGGTATTGCAACACTCCC
#42	3' (1.0)	F	AActcgagGTGGGCGTTGTCCTGCAGGG
#42	3' (1.0)	R	AAggtaccCAAGCTCACAAGACCTTAGG

Red-colored letters indicate point mutations and/or FLAG sequences.
Small letters indicate the restriction enzyme sites.

Supplementary Table S6. Oligonucleotides used for genotyping PCR

gene			sequence (5' to 3')
#22-EGFP	5'	F (c)	ATGTAACAATACAGTGGATTCTCC
		R (d)	TCACCTTGATGCCGTTCTTCT
	3'	F (e)	GCCACCATGGTGAGCAAGGGCGAG
		R (f)	AGATGACCTGGAGTTATGTAAGCC
#42-zeocin	5'	F	CTCAGAGAGCCTCGGCTAGG
		R	AACGGCACTGGTCAACTTGG
	3'	F	CAACTGCGTGCACTTCGTGG
		R	AGAATGCCATGAGTCAAGCC
Gapdh (control)		F	AGTGGAGATTGTTGCCATCAACGAC
		R	GGGAGTTGCTGTTGAAGTCGCAGGA

Supplementary Movies S1 and S2. Motility of tailless *Dnajb13* KO spermatozoa

The DIC image and GFP fluorescence of the *Dnajb13* KO spermatozoa were shown in Movies S1 and S2, respectively.

Supplementary Movies S3 and S4. Motility of short-tailed *Dnajb13* KO spermatozoa

The DIC image and GFP fluorescence of the *Dnajb13* KO spermatozoa were shown in Movies S3 and S4, respectively.
